# The Need for Parental Support for Migrant Parents in Transition Into Sweden: A Perspective

**DOI:** 10.3389/fpubh.2022.680767

**Published:** 2022-04-29

**Authors:** Elisabeth Mangrio, Karin Enskär, Rathi Ramji, Katarina Sjögren-Forss, Per-Anders Tengland, Kyriakos Theodoridis, Slobodan Zdravkovic, Margareta Rämgård

**Affiliations:** Department of Care Science, Faculty of Health and Society, Malmö University, Malmö, Sweden

**Keywords:** parental support, migrants, child health care, Sweden, culturally acceptable

## Abstract

Migration is a stressful experience and research shows that newly arrived migrants in Sweden suffer from different challenges and struggle to relate to parenting in a new culture that is different from their own. The Swedish Child Health Services (CHS) focuses on promoting health among children, as well as supporting parents in parenting. Although this is a goal, migrant parents participate at lower rates in parental support groups. This paper aims to discuss how the Swedish CHS can support these families and address the need for improvement in the parental support offered to migrant parents during transition into their host country. In addition, this paper also aims to review and discuss the advantages of using a community-based participatory research approach together with the Swedish CHS to identify and apply culturally appropriate support programs to increase health literacy among migrant parents. The Swedish government decided to place greater emphasis and resources on supporting parents and promoting equal health among families in Sweden, with special emphasis on migrants and other vulnerable groups. This report from the Swedish government indicates the importance of creating knowledge about new ways, methods, and actions that may be needed to increase this support. One suggestion of this paper is to provide culturally appropriate healthcare work using a community-based participatory research approach, where migrant parents themselves are actively involved in the development of support programs. This approach will not only provide migrant families knowledge and support, it will also build on their needs and the challenges they can share, and receive support to overcome.

## Introduction

Migration is a stressful experience (1) and research shows that migration-related events can contribute to stress (2). After migration and arrival in a new country, the migrant usually must wait for the asylum process to proceed, and this wait can worsen the mental health of some migrants (1, 3). Sweden has historically been a welcoming and recipient country for migrants with a refugee background (which we in this current paper address as migrants) and during 2015 around 160,000 migrants applied for asylum as refugees in Sweden (4). During 2020, there was a decrease in the number of refugees entering in Sweden, due to the pandemic and only 13,000 refugees applied for asylum that year. The countries from where the refugees are coming from have differed during the years. In the beginning of 1990, mainly refugees from ex-Yugoslavia were applying for asylum due to the war situation there and during 2015, Syria was highly representative among new-coming refugees in Sweden (4). During 2020, Syria followed by Uzbekistan and Iraq had the highest numbers of refugees entering Sweden (4). Sweden has an establishment program for all newly arrived refugees attaining refugee status in Sweden and this is a 2-year program containing Swedish studies, society and health communication and activities for getting into the employment market in Sweden (5, 6). When refugees are active within this program, they are entitled to receive economic supply for themselves and their children (5). During the last years, there have been some restrictions on refugees' rights to receive their partners and children into the country and if they should unite as families, the newly arrived migrant in Sweden has to ensure financial support for the whole family (7). New research from the research-based platform of migration and health shows that newly arrived migrants in Sweden suffer in various ways after arrival (8, 9). For example, many families suffer from challenges concerning living conditions, and others experience mental suffering due to separation from family or extended family (10). New qualitative research in Sweden concerning migrant families' situations indicate that they also struggle with parenting in a culture different from their home countries' (10). Another Swedish study on refugees and health, shows that refugees are suffering to a high proportion of mental ill-health and low quality of life (11). During the recent pandemic, it has been evident that migrants residing in Sweden, have been suffering to a higher degree related to COVID-19, unmet healthcare needs and it has increased their social vulnerability (12). According to Samarasinghe (13), migrant families face a complex transition when they try to navigate and establish themselves in the host country. This research points to the importance of nurses working closely with migrant families, and the need for nurses to understand the socio-contextual environment in which migrant families live, in order to be able to help them navigate this process. Moreover, Samarasinghe claims that when families originating in collectivistic-oriented cultures seek refuge in individualistic-oriented cultures, which emphasize the autonomy of the individual, migrants risk interpersonal conflicts due to a change in the nature of cultural values and family roles in the host country (13). Recent research on parenting among asylum seekers in Sweden shows that these parents were reporting that they felt like they had few rights, had concern about practical issues such as money and housing, had a constant fear of being repatriated and these factors increased the mental ill-health among these parents as well as among their children (14). Since we know the complex situation that migrants face when transitioning into a new country and the challenges that they can face as a family (10, 13), it is important to highlight and evaluate the support that Swedish Child Health Services (CHS) can give these families. Therefore, this paper aims at discussing how the CHS can support these families and address the need for improvement in the parental support that is offered migrant parents during their time of transition into the host country. In addition to that, the paper also aims at looking at this support program from a community-based participatory research approach and with the aspect of how important health literacy is when aiming at reaching and supporting migrants.

## CHS Parental Support

Families with children between 0 and 5 years of age are most often regularly attend the Swedish CHS (15). And all children residing either legally or illegally are eligible for CHS service which is also free of charge. CHS is a well-established organization with the aim of reducing morbidity, mortality and disability among children between 0 and 5 years (14, 15). Through regular visits to the CHS, children's development is closely monitored and vaccinations are also given (15). The primary goal of the CHS is to promote equal health and prevent diseases among children. To achieve this goal, the CHS aims to offers support not only to children but also to parents so as to enhance their parenting skills which may further help create favorable conditions for the overall development of the children. An additional focus is also set on providing additional support particularly to parents and children in vulnerable and disadvantaged situations such as migrants who have a higher risk of developing ill health or already experience impaired health (16, 17). This goal of working toward equal health for all children is also emphasized by the UN Convention on the Rights of the Child (UNCRC), ratified in 1989 (18), of which Sweden was one of the first signatories (19). This treaty states the rights for all children, including migrant children in Sweden, regardless of their respective families' residency status. Although the CHS in Sweden has this goal, the parental support program that is offered to all parents during the child's first year does not reach everyone, since not all parents are willing to participate in these programs (16). It has been observed that migrant parents and parents with lower levels of education are especially hard to reach, and attend meetings with CHS nurses at lower rates (16). There are other examples of migrants being difficult to reach within CHS and one such example is screening for post-partum depression. Research related to screening for post-partum depression among migrant women in Sweden show that they are not offered screening to the same proportion as the rest of the women and that they do not agree to participate to the same extent as the other women in Sweden (20, 21) even if the proportion of migrant women that suffer from post-partum depressions are higher than the rest of the population (22). Prior studies have also shown that untreated post-partum depression may have negative consequences on health of both mothers and children. Prolonged post-partum depression in mothers which remains untreated has been shown to affect cognitive functioning among young children, while adolescents in the household have also seen to develop violent behavior, as well as psychiatric and medical disorder. In order to prevent these adverse problems, the CHS has taken measures to offer early and effective parental support to migrant parents, which often do not reach the mothers given cultural and contextual barriers.

Several Swedish studies conclude that there is a need for improvement regarding the cultural competence (knowledge, skills, etc.) among nurses working within CHS (23). This makes it difficult for them to adjust healthcare to families' needs (24–26). Cultural competence is required and crucial, since research shows that migrants suffer to a high degree from post-traumatic stress syndromes (27), which in turn might affect their parenting ability, but also require understanding from nurses that engage with them. Moreover, in a study involving Somalian migrants in Sweden, regarding their perceptions of their need for support in their parenting roles (28), parents described needing information on how to culturally adapt and improve their parenting, as well as obtain support from the authorities.

In 2018, the Swedish government placed greater emphasis and resources on supporting parents, aiming, through with a support program, to increase health equality among families in Sweden, with a special emphasis on groups such as migrants, that could be in need of extra support (29). This governmental initiative highlights the importance of acquiring knowledge about new means, methods and actions to improve support, in order to reach equality of health among all families (29).

We can conclude, then, that in general, migrants lack trust in societal institutions and people working in them (8, 9). However, among migrants, there is a high level of trust for the CHS (8, 9). We will argue below that this trust should be built on in working with migrant parents in Sweden. Although, based on these discussions it is clear that this support program is a promising initiative, there is a need for knowledge regarding what the support program should include and how they can be adapted to the actual needs of the parents.

## The Aspect of Health Literacy Among Migrants

Another important aspect relating to child health care for migrants in Sweden is health literacy. Health literacy refers to the ability of individuals to acquire and understand basic health information and instructions in medical and healthcare contexts, together with the ability to access crucial health services and support systems. It therefore concerns an individual's ability to make decisions about their own health, and their family's (30, 31), and it is crucial for their empowerment (32). Health literacy amongst migrants has generally been found to be low (33) and associated with overall poor health, sub-optimal experiences with healthcare, and a tendency to abstain from seeking healthcare (34). A large part of this challenge arises from language difficulties, which result in this subgroup being ill-informed about how the healthcare system works (35), unaware about their healthcare entitlements (36), and often confused or distrustful following experiences where they feel that nobody is taking responsibility for their care, as when they are sent to different healthcare units without sufficient assistance or guidance (37). Health literacy is an important determinant of health and closely related to health inequalities (38). With health inequalities growing and seemingly becoming the norm rather than the exception in Sweden and other European countries (39), it has become imperative to address and eliminate health inequalities through a range of initiatives, such as increasing health literacy among migrant groups. In particular, it is important to consider this when planning new ways of working with migrant parents in order to ensure them parental support in a culturally appropriate way and to ensure not only that the migrants understand but that they are also are able to apply the information and support that they receive from these kinds of programs. A recently published research also highlights the increasing need to focus on elevating health literacy especially during the acute situations as the recent pandemic, to ensure that migrants understand as well as can access the support and care they need during these times (40). Another Swedish study on health literacy among refugees emphasizes the need for health literate health organizations that can meet the challenges that refugees face and further also suggests that health-professionals must give more importance to the aspect of health literacy (41).

## New Methods When Working With Migrants

Research shows that health programs and interventions for health literacy are more effective when they are culturally appropriate (42) and that, when they are culturally appropriate, that these programs and interventions could improve migrants' access to information and possibilities for enhanced health literacy (42). It has been a persistent problem that migrants lack trust in the Swedish health care system for reasons that include differences in structure and practice from the healthcare systems of their native countries (12, 43). Migrant health care support programmes seem to have little or no success since the clinical staff within the health care systems fail to understand the unique health and social needs of migrants, have the knowledge and skills to motivate them to the value of these support programs (44). One solution to this problem, is that researchers work together in an equal partnership with the parents and stake holders from CHS with a goal to define the problem and identify collaborative interventions for families to better support theirs needs during their transition in the host country (45, 46). Community-based participatory research (CBPR) is a collaborative effort, where knowledge is co-created in this context, given that it involves engaging immigrants not only in identifying their own problems, but also to resolve them together with concerned stakeholders such as healthcare professionals (47). CBPR is a model for building on the strength and resources of communities through connecting different stakeholders to citizens, and working collaboratively to achieve health-related community goals. In contrast to traditional systems, where citizens approach healthcare, in the CBPR-approach health providers, together with other stakeholders from the public, private, and non-profit sectors, reach out to the citizens in their own environments, in order to identify and resolve the health-related challenges of the communities in partnership with them (48). Such a method facilitates the creation of a “neutral” environment to co-create knowledge in otherwise neglected communities and enhance their ability to autonomously take control over their health and well-being (49). This emphasis on acquiring ability and autonomy is connected to a general idea and ideal, namely, that all citizens should be empowered to take control over their lives (50). Previous research with migrant families from disadvantaged neighborhoods used a participatory health approach, using dialogue-based teaching where families participated in reflective dialogue sessions facilitated by professionals in relevant fields, and, in doing so, addressed challenges that emerged from the very needs of the community (51). The CBPR approach is inspired by Brazilian pedagogue Paulo Freire's “culture circles,” where the aim is to nurture a participatory experience with a focus on dialogue and reflective action following an emancipatory health education (52). This approach rests on the assumption that actions taken for social change to improve community health should start from building on the strengths of communities through involving them equitably in the process of knowledge mobilization (52).

The crucial step in this approach is trust building, as described in the above-mentioned study (51), especially given the migrants' mistrust in the healthcare system and even in academia. Trust building promotes prolonged engagement, which leads to increased social support and positive peer influence within the group. As part of this previous study, healthcare staff, including dieticians, nurses, and dentists, initiated health-educational sessions with interactive presentations, followed by dialogic exchange, experience sharing, and reflection processes, with migrant families (51). The results of focus-group evaluations made with families following these reflective dialogue sessions showed that the interactive dialogues and discussions promoted health-related changes among these socially disadvantaged immigrant families. In addition, the families perceived that these sessions were more informative and practically useful, given that they were adapted to their needs compared with the short encounters with personnel in primary care centers or at dental clinics, which were most often irrelevant, since the advice or information provided, e.g., in pamphlets, were adapted to a general Swedish audience (51). Another important finding is that this method's multi-stakeholder perspective, where professionals work in partnership with local communities, has the potential to build trust between the community and the healthcare system (51).

CBPR has been used in the context of health promotion particularly among migrants for over three decades given that it primarily focuses on problems regarded by the communities themselves with the aim of combining knowledge and action to bring about social change and further to facilitate integration, improve health of the community members and there by contribute to reducing health inequalities (47, 53). Aside of its application in public health and health promotion, CBPR has also been widely used by primary care practioners in the past decade to address common health problems, noticed in primary care such as perceived access to care and disparities in chronic disease management among vulnerable groups including migrants (45, 54). Although CBPR approach has not been previously applied in the context of promoting health literacy by child health care services elsewhere, it has been proven effective in parental educational programs aiming to improving immunization rates among immigrant children in the United States (55). Further, several community-based parental web programs to promote parenting skills among Latino migrants in the United States developed and implemented using a CBPR approach showed that the program not only promoted power sharing and building trust among community members but also enhanced parents adherence to the program (56).

## Discussion

Migrant parents face a complex situation and challenges both for themselves and their children when trying to navigate in a new country, and during this transition it is important for health professionals working and engaging with these families to know and understand that complex situation and the challenges they face. Although the CHS has set a goal of reaching all parents with a parental support program, we know that migrant parents participate at lower rates. Even if this is the case, migrants have shown high levels of trust in the CHS compared to other societal institutions or healthcare settings (8, 9). This trust is a foundation that can be built upon. One reason for this trust might be the continuity of care often seen within CHS, where the same nurse meets specific families over time. Continuity of care, then, might be one general way to better succeed in reaching and supporting migrant families with or without children. This could work as a model for other countries with similar high reception of migrants and with needs of adapting child health care to suit the needs of migrant groups that could enter a country.

For example, a study among migrant parents from Somalia residing in Sweden showed that the parents perceived a need for information on how to culturally adapt and improve their parenting (28). Moreover, it was found that it is important that interventions offered to migrant parents are also culturally adapted in order to reach them and meet their needs (57). Given that Somalians are one of the migrant groups in Sweden there is a need for similar studies with other migrant groups in Sweden, since it has emerged from other studies that migrant parents have believed that parenting is challenging in a country with a culture quite different from their own (10, 28). This also goes for addressing sensitive sexual health information (29), where the information provided is preferably provided by health communicators with backgrounds similar to those of the newly arrived (58). Thus, it is important to provide culturally adapted services and, if possible, facilitated by a multidisciplinary team where professionals work in close collaboration with select members of the community known as lay health promoters or brokers from similar backgrounds (59, 60) when working with sensitive health issues. This suggests the need for community engaged and participatory approaches in educational programs that are intended for migrant groups.

Moreover, several sources have highlighted the importance of working in collaboration with parents and families. The International Society for Social Pediatrics and Child Health (61) have emphasized the importance of listening to the migrants regarding child health care. They also emphasize the need for working closely with other sectors of society when engaging with migrant families (61). Such collaborations might be one further means of culturally adapting the support program to the needs of migrant parents in Sweden.

This discussion paper has raised one possible way of working collaboratively with families to achieve a goal of promoting health by using the CBPR approach that has been successfully applied in a Swedish neighborhood predominated by families of migrant background (51). The model helps facilitate the creation of a safe environment where the migrant families can express themselves more readily and, with support from healthcare professionals and other stakeholders, can possibly enhance their empowerment, and therefore also their ability to take responsibility for their family's health and well-being (49). This approach, then, seems to be a way of not only providing migrant parents and families with what health professionals and researchers believe and assume they need, but to build upon these parents' and communities' perceptions of their needs. This is also much in line with the recommendation of The International Society for Social Pediatrics and Child Health (61). Yet another advantage of working with a CBPR approach is that researchers involved in the development and implementation of the intervention continuously study the processes initiated. This means that the results of the research conducted will, while facilitating families to take control over their health and lives, also create more knowledge about how such change processes evolve and can be improved as well as inform its application in other similar settings.

Engaging migrant parents in the research process can be challenging, however there are several approaches to address these challenges including initiating a trust-building process so as to initiate the establishment of equitable and sustainable partnerships with the community, therefore we propose the use of lay health promoters to reach out to these parents and work with the CHS and the researcher within the research process. Furthermore, migrant parents often perceive language barriers that may hinder communication during the research process that may also motivate their disengagement from such initiatives, however the use of lay health promoters also as interpreters ensures effective communication between the parents and the societal stakeholders including those in CHS.

Furthermore, CBPR relies on power sharing structure for joint partnership. In the child health care, the professionals have a certain level of interpretive precedence which limits their understanding of the actual needs of the parents and CBPR approach can neutralize such power imbalances. The CBPR suggest a bottom-up approach where power is said to be balanced in the partnership between health care personnel and the communities, although challenges may occur owing to shift of power; and changing the traditionally constructed organizational system. Swedish health and social care are vertically organized institutions with a top-down approach where the decisions are already made at the top levels thus ignoring the crucial input of the communities that benefit from these decisions (62, 63).

To sum up, in the proposed approach lay health promotors act as bridges between the CHS personnel and the migrant parents, helping to establish trust in the partnership, while also communicating the cultural nuances of the community to the health care staff. The proposed work also does not demand large organizational decisions that may be time consuming to arrive at, but more a change in work context and approach to health promotion among individual professional in CHS at a local level working in collaboration with the academic researchers.

## Conclusion

Future work by healthcare professionals aiming to provide parental support to migrant parents in Sweden should aim to take an inclusive approach, such as that of CBPR, so as to maximize the potential effect of the support programs to promote health among migrant children.

## Data Availability Statement

The original contributions presented in the study are included in the article/supplementary material, further inquiries can be directed to the corresponding author.

## Author Contributions

All authors listed have made a substantial, direct, and intellectual contribution to the work and approved it for publication.

## Funding

Open access fee was funded by the library at Malmö University, Sweden.

## Conflict of Interest

The authors declare that the research was conducted in the absence of any commercial or financial relationships that could be construed as a potential conflict of interest.

## Publisher's Note

All claims expressed in this article are solely those of the authors and do not necessarily represent those of their affiliated organizations, or those of the publisher, the editors and the reviewers. Any product that may be evaluated in this article, or claim that may be made by its manufacturer, is not guaranteed or endorsed by the publisher.
